# Ecology Careers in the AI Era: Early‐Career Data Literacy Must Be Protected

**DOI:** 10.1002/ece3.73672

**Published:** 2026-05-17

**Authors:** Mohamed Khalil Meliane, Erin L. Koen, E. Hance Ellington

**Affiliations:** ^1^ Range Cattle Research and Education Center University of Florida Ona Florida USA; ^2^ Independent Researcher Bradenton Florida USA; ^3^ Department of Wildlife Ecology and Conservation University of Florida Gainesville Florida USA

**Keywords:** AI literacy, artificial intelligence, automation, ecology workforce, entry‐level positions, machine learning

## Abstract

The rapid adoption of artificial intelligence (AI) in ecology signals a shift in how organizations structure entry‐level positions and the role of young ecologists in data processing tasks. Implementing AI to automate data processing could mean that stepping‐stone positions provide less training of key skills needed for career growth. To aid educators, employers, and early‐career ecologists in navigating the adoption of AI into ecology and conservation workflows, we outline two plausible paths for the structure of entry‐level positions: one where data processing and management are streamlined by AI and the entry‐level ecologist focuses almost exclusively on hands‐on fieldwork, and a second path where, in addition to fieldwork, entry‐level ecologists participate in the implementation of AI‐assisted data processing tasks. We argue for the second path, the analyst‐technician model, and recommend that employers reserve workday time to involve mentees in data processing pipelines using novel technologies to protect training, broaden participation, improve career persistence, and keep early‐career ecologists competitive for the next steps in their careers.

## Abstract 2

The rapid adoption of artificial intelligence (AI) in ecology signals a shift in how organizations structure entry‐level positions and the role of young ecologists in data processing tasks. Implementing AI to automate data processing could mean that stepping‐stone positions provide less training of key skills needed for career growth. To aid educators, employers, and early‐career ecologists in navigating the adoption of AI into ecology and conservation workflows, we outline two plausible paths for the structure of entry level positions: one where data processing and management are streamlined by AI and the entry‐level ecologist focuses almost exclusively on hands‐on fieldwork, and a second path where, in addition to fieldwork, entry‐level ecologists participate in the implementation of AI‐assisted data processing tasks. We argue for the second path, the analyst‐technician model, and recommend that employers reserve workday time to involve mentees in data processing pipelines using novel technologies to protect training, broaden participation, improve career persistence, and keep early‐career ecologists competitive for the next steps in their careers.

## 1 Introduction

Across industries, entry‐level positions seem to be the most vulnerable to the adoption of artificial intelligence (AI) (Ghosh et al. 2025, Lichtinger and Hosseini Maasoum 2025). In ecology and conservation, the impact of AI on common entry‐level roles, such as wildlife technicians, junior consultants, or assistant ecologists (hereafter called technicians), can be less obvious because these roles commonly blend fieldwork with basic data processing. For example, a wildlife technician working on a project monitoring wildlife diversity might go into the field to maintain a network of motion‐triggered cameras and in the same day scan through photos, identify the species captured in each image, and enter that information into a database. Emerging AI tools are now automating tasks such as identifying wildlife in camera images or bird calls on audio recordings, and supporting remote sensing‐based habitat mapping and vegetation classification, with the promise of making data available for analysis quicker, cheaper, and with fewer biases (Oba and Doi 2025; Lansu et al. 2025; Barnas and Fisher 2025). Because many ecology‐specific AI tools are still young compared to those in other industries (e.g., Gadot et al. 2024, Čermák et al. 2024, Otarashvili et al. 2024; see Gewin 2025 for a recent overview), empirical clarity on their potential career impacts on entry‐level positions in ecology is limited. And while routine data‐processing tasks are within the reach of current automation technology, fieldwork tasks such as checking live traps and handling animals, deploying and maintaining monitoring equipment, or conducting fine‐scale habitat surveys, remain comparatively human‐dependent (Si‐Moussi et al. 2025). The field‐based tasks that current AI tools cannot perform have created an apparent shelter for ecology professionals, delaying an urgent discussion: how will AI reshape early‐career training and the ecology career ladder?

## 2 Entry‐Level Positions Are More Impacted

A recent report by the US National Bureau of Economic Research argued that the number of jobs lost to advancements in AI is balanced by AI‐enabled growth and increased labor demand (Hampole et al. 2025). However, those gains and losses in employment do not necessarily affect the same rung of the career ladder. While demand for more experienced employees is rising, initial reports indicate that there are fewer entry‐level opportunities in industries that have adopted AI (Fedyk et al. 2022, Lichtinger and Hosseini Maasoum 2025).

The impact of fewer entry‐level positions is compounded by challenges to advancement up the career ladder if AI automates the data‐processing tasks that were previously a part of entry‐level career training. Bailey et al.'s (2022) analysis of individual career success in ecology showed that young professionals that acquire training in data management and analysis are more competitive for the next career step and see higher long‐term success relative to their more field‐experienced peers. Thus, unmanaged AI adoption in ecology could further reduce opportunities for early career wildlife ecologists to develop data skills, limiting marketability and hampering future career success.

Critically, the ongoing shift in the organizational hierarchy and the decline of entry‐level positions create a system where young professionals may face opposing, concurrent challenges. Ecology entry‐level roles will likely offer less hands‐on time with data as senior staff process data using AI‐streamlined pipelines (Norouzzadeh et al. 2018; Barnas and Fisher 2025). Concurrently, senior‐level positions will require more data literacy, with candidates expected to demonstrate fluency with AI and other emerging technologies. In these situations, candidates would be advancing up the career ladder from entry‐level roles that did not equip them with the skills needed to perform such tasks. It is our view that facing these shifts, supervisors of entry‐level ecologists aiming to support their mentees' long‐term career success should purposefully aim to expand the positions' involvement in data processing.

## 3 Choose the Right Path

Although some of our examples draw from academic research, these trends are likely mirrored across ecology and conservation sectors, including commercial consulting, government agencies, and nongovernmental organizations, where entry‐level roles may be titled differently but still combine fieldwork with desk‐based and data‐processing tasks. Across sectors, the rapid incorporation of AI into ecology workflows will likely lead entry‐level positions down one of two plausible paths.

### 3.1 Path A: All Field, Little Data

In one scenario, as AI streamlines data processing and management, employers stop budgeting time for their entry‐level staff, such as wildlife technicians, to perform data processing tasks. Instead, they justify salaries for technicians only where human observers are indispensable, such as for trap checks, animal capture and handling, on‐site habitat measurements, or plant surveys. A technician might spend most days driving to sites, servicing equipment, collecting samples, recording observations in the field, and assisting with field logistics, while having little involvement in how data are later cleaned, validated, stored, or analyzed. While to employers this would likely be the most financially efficient path in the short term, a field‐only pathway narrows training, cutting early‐career professionals off from emerging analytical tools, data stewardship practices, and model‐evaluation skills—competencies that will be necessary to progress into senior‐level positions. Additionally, centering entry‐level positions exclusively around fieldwork may worsen access barriers for groups of aspiring ecologists, including people with disabilities and candidates facing financial or logistical constraints (Morales et al. 2020, Chasen et al. 2025).

### 3.2 Path B: Analyst‐Technician Roles

In an alternate scenario, a technician's time is split between field and office. A technician's day might consist of mornings in the field (e.g., deploying sensors, checking live‐traps and handling animals, or troubleshooting equipment), with afternoons spent in the office on AI‐assisted labeling, auditing model accuracy, curating datasets, improving metadata, maintaining databases, and preparing files for downstream analyses. Technicians might also help compare AI outputs against human observations, flag ambiguous records, participate in quality control decisions, and assist with data workflows. Here, AI becomes a teaching tool: technicians learn to verify outputs, quantify uncertainty, and gain a practical understanding of the capabilities of current technologies. This pathway will best support career growth for entry‐level professionals because it preserves the training function of their positions in the changing technological landscape.

The feasibility of *Path B* will, however, vary across employers and sectors. Seasonal positions, salary structures, training time, and differences in how work is funded may limit how much time organizations can devote to skill development in the short term. Yet, excessively narrowing entry‐level roles risks weakening the training and recruitment pipeline into more experienced positions by reducing opportunities to build the data literacy and professional competencies needed for career progression. Realizing this path will require both planning and investment, not only by early‐career ecologists but by all parties involved in their training.

- Postsecondary or tertiary ecology curricula should adapt coursework and training to include data management, reproducible workflows, and applications of AI and machine learning. Discussion of the tools and their limitations can help students enter the workforce with a better understanding of emerging technologies.
- Early career ecologists should adopt a long view of their career development, prioritizing positions with integrated field responsibilities and data work that will equip them with transferable skills.
- Employers should intentionally involve early‐career ecologists in automated processes and train them on novel tools and how to use them. They should integrate field protocols with data skills and develop training plans for technician positions to provide their mentees with the experience they need to benefit from rapid technological advancement.
- Funders and sector bodies should consider future‐facing mentorship and capacity‐building as measurable project goals. Salary lines that include concrete data‐literacy outcomes should be recognized as workforce investments. An alternative model where young ecologists are expected to learn data management and analysis on their own time limits participation and equitable access, especially for candidates without financial buffers (Fournier and Bond 2015).

## 4 Automation Is Attractive and Beneficial, but Let's Keep Early‐Career Training in Mind

In ecology, the benefits of AI in terms of the efficiency of data processing, pattern detection, and decision‐making support are undeniable (Reynolds et al. 2025). AI‐assisted processing of large datasets and increasingly automated workflows boost productivity and support more responsive, near‐real‐time ecological monitoring and decision‐making (Davison et al. 2025; Kershenbaum et al. 2025). Ecologists have embraced previous waves of automation in their workflows, such as the widespread adoption of automated sensors (e.g., remote sensing) to collect ecological data, which have enhanced our capacity to monitor ecosystems but are not without consequences (Soga and Gaston 2025). Now that automation is reaching for data processing, it also threatens increasingly essential skills for young ecologists. AI technologies are accelerating and investment into the analyst‐technician roles will protect the training function of entry‐level jobs, broaden participation, improve research quality, and produce a more adaptable, inclusive ecology workforce that is better equipped for future technological change.

## 5 Conclusion

Current and emerging technologies are likely to change the structure of entry‐level ecology positions. The risk is that these positions become narrower and less formative while data literacy, AI literacy, and workflow oversight skills become more important for career advancement. Protecting opportunities for early‐career ecologists to participate in both fieldwork and data processing pipelines will help ensure that AI strengthens ecological research without diminishing the training of the next generation.

## Author Contributions

**Mohamed Khalil Meliane:** conceptualization (lead), investigation (lead), writing – original draft (lead), writing – review and editing (equal). **Erin L. Koen:** conceptualization (equal), investigation (equal), writing – original draft (equal), writing – review and editing (equal). **E. Hance Ellington:** conceptualization (equal), investigation (equal), writing – original draft (equal), writing – review and editing (equal).

## Funding

The authors have nothing to report.

## Conflicts of Interest

The authors declare no conflicts of interest.

## Data Availability

The authors have nothing to report.
